# Ameliorating effects of L-carnitine and synbiotic co-supplementation on anthropometric measures and cardiometabolic traits in women with obesity: a randomized controlled clinical trial

**DOI:** 10.3389/fendo.2023.1237882

**Published:** 2023-10-18

**Authors:** Farnoush Fallah, Reza Mahdavi

**Affiliations:** ^1^ Student Research Committee, Nutrition Research Center, Tabriz University of Medical Sciences, Tabriz, Iran; ^2^ Nutrition Research Center, Department of Biochemistry and Diet Therapy, Faculty of Nutrition and Food Sciences, Tabriz University of Medical Sciences, Tabriz, Iran

**Keywords:** L-carnitine, multi-strain/multi-species synbiotic, concomitant supplementation, anthropometric indices, lipid profile, glycemic parameters, cardiometabolic biomarkers, obesity

## Abstract

**Background:**

Obesity, a multifactorial disorder with pandemic dimensions, is conceded a major culprit of morbidity and mortality worldwide, necessitating efficient therapeutic strategies. Nutraceuticals and functional foods are considered promising adjuvant/complementary approaches for weight management in individuals with obesity who have low adherence to conventional treatments. Current literature supports the weight-reducing efficacy of pro/pre/synbiotics or L-carnitine; however, the superiority of the nutraceutical joint supplementation approach over common single therapies to counter obesity and accompanying comorbidities is well documented. This study was designed to assess the effects of L-carnitine single therapy compared with L-carnitine and multistrain/multispecies synbiotic co-supplementation on anthropometric and cardiometabolic indicators in women with obesity.

**Methods:**

The current placebo-controlled double-blind randomized clinical trial was performed on 46 women with obesity, randomly allocated to either concomitant supplementation [L-carnitine tartrate (2 × 500 mg/day) + multistrain/multispecies synbiotic (1 capsule/day)] or monotherapy [L-carnitine tartrate (2 × 500 mg/day) + maltodextrin (1 capsule/day)] groups for 8 weeks. Participants in both groups received healthy eating dietary advice.

**Results:**

Anthropometric, lipid, and glycemic indices significantly improved in both intervention groups; however, L-carnitine + synbiotic co-administration elicited a greater reduction in the anthropometric measures including body mass index (BMI), body weight, and neck, waist, and hip circumferences (*p* < 0.001, <0.001, <0.001, = 0.012, and =0.030, respectively) after adjusting for probable confounders. Moreover, L-carnitine + synbiotic joint supplementation resulted in a greater reduction in fasting blood sugar (FBS), insulin (though marginal), and homeostatic model assessment of insulin resistance (HOMA-IR) and more increment in quantitative insulin sensitivity check index (QUICKI; *p* = 0.014, 0.051, 0.024, and 0.019, respectively) compared with the L-carnitine + placebo monosupplementation. No significant intergroup changes were found for the lipid profile biomarkers, except for a greater increase in high-density lipoprotein-cholesterol concentrations (HDL-C) in the L-carnitine + synbiotic group (*p* = 0.009).

**Conclusion:**

L-carnitine + synbiotic co-supplementation was more beneficial in ameliorating anthropometric indices as well as some cardiometabolic parameters compared with L-carnitine single therapy, suggesting that it is a promising adjuvant approach to ameliorate obesity or associated metabolic complications through potential synergistic or complementary mechanisms. Further longer duration clinical trials in a three-group design are demanded to verify the complementary or synergistic mechanisms.

**Clinical trial registration:**

www.irct.ir, Iranian Registry of Clinical Trials IRCT20080904001197N13.

## Introduction

Obesity, a multifactorial relapsing chronic disease attributed to the complicated interaction of behavioral, environmental, and genetic factors (1), is well recognized as a prominent driver of morbidity and preventable mortality worldwide (2, 3). The global prevalence of obesity has dramatically amplified in recent decades, reaching pandemic dimensions (4), warranting efficient therapeutic approaches. Lifestyle and dietary modifications are the cornerstone of therapeutic strategies for weight reduction (5); however, adherence to weight-reducing programs or sustaining lifestyle changes is a frustrating challenge for many people (6). Given the adverse effects of anti-obesity medications, there has been a great appeal in the consumption of weight loss supplements among individuals suffering from obesity seeking a “magic bullet,” which is less demanding than conventional weight management protocols (7). Accordingly, the consumption of weight loss supplements, particularly nutraceuticals/functional foods, as adjuvant/complementary therapies has attracted great attention.

In this context, L-carnitine, a conditionally essential nutrient (8) performing a crucial role in glucose and lipid metabolism (9, 10), has been widely consumed as a popular over-the-counter (OTC) weight loss supplement, due to its purported health-beneficial properties including potential anti-obesity (11, 12), antidiabetic (13), and lipid-improving effects (14, 15). Evidence has reported the weight-reducing effects of L-carnitine (11, 12); additionally, previous studies demonstrated the improving effects of L-carnitine on cardiometabolic risk factors including lipid (14, 16) and glycemic indexes (13, 17), possibly through contributing in fatty acid ß-oxidation (8, 18); increasing energy expenditure via modulating the acetyl-CoA/CoA ratio (19), thus improving insulin sensitivity and activating the glycolytic pathway (8, 10); or stimulating adipocyte lipolysis, as well as reducing adipogenesis in adipocytes, through modulating lipolytic/adipogenic gene expression (20, 21).

Furthermore, the crucial role of gut microbiota dysbiosis as an underlying driver of obesity has attracted great concern (22–24). According to evidence, metabolic disturbances including obesity and related complications are correlated with alterations in gut microbiota diversity and composition (23). Consequently, gut microbiota is recognized as a promising therapeutic goal to ameliorate dysbiosis-generated metabolic disorders specifically obesity (25). Therefore, evaluating the efficiency of microbiota-remodeling strategies, predominantly pro/pre/synbiotic therapy, on obesity and metabolic comorbidities has come into focus (24, 25). The weight-reducing effects of pro/synbiotic supplementation were reported in a couple of meta-analyses (26, 27). Likewise, another meta-analysis revealed an improved lipid profile following synbiotic administration (28); also, synbiotic therapy diminished fasting insulin and triglyceride concentrations (29). Several potential mechanisms are proposed for the ameliorating impacts of pro/pre/synbiotics on anthropometric/metabolic indices including, but not limited to, modulating gut microbiota dysbiosis, thereby decreasing adipogenesis and enhancing lipid oxidation (30); augmenting the production of short-chain fatty acids (SCFAs) production (31–33), thus amending energy homeostasis and fat storage via boosting fatty acid oxidation (30); ameliorating glucose homeostasis and mitigating insulin resistance (31, 33); and ultimately attenuating gut permeability and metabolic endotoxemia, thereby hindering proinflammatory signaling pathways (25, 31, 34, 35).

According to literature, multistrain synbiotics are reported to be more beneficial in modulating gut microbiota than single-strain synbiotics or pro/prebiotics individual therapy (36–38). Moreover, existing evidence supports the advantage of the nutraceutical joint supplementation approach to counteract obesity and accompanied disorders, compared with conventional monotherapies (39). Accordingly, previous studies have reported that co-administration of L-carnitine concurrent with common pharmaceutical/nutraceutical therapies (e.g., orlistat, sibutramine, genistein) might confer greater improving effects on either weight/BMI, glycemic, or lipid profile, vs. individual administration of each medication (40–42), which has been ascribed to the additive or synergistic impacts of combined therapy.

As mentioned, current data indicate the putative efficacy of both L-carnitine (11–15) and pro/pre/synbiotics in ameliorating obesity and associated metabolic (lipid/glycemic) indicators (26–29). Furthermore, it has been supposed that L-carnitine combined therapy might be more efficient in attenuating obesity and related comorbidities compared with conventional monotherapies (40–42). Additionally, the possible synergistic/complementary impacts of L-carnitine and pro/synbiotics (43) should be considered. Therefore, the simultaneous supplementation of synbiotics and L-carnitine for concurrent targeting of diverse metabolic pathways presents a promising and reasonable approach to alleviate obesity. Hence, we hypothesized that concomitant supplementation of multistrain/multispecies synbiotics and L-carnitine may induce more pronounced effects on weight or metabolic parameters. Nevertheless, we found no previous reports on the metabolic effects of synbiotics and L-carnitine co-administration in individuals with obesity. Therefore, the present research was conducted to assess the effects of concomitant supplementation of L-carnitine and a multistrain/multispecies synbiotic compared with L-carnitine single therapy on the anthropometric and cardiometabolic indices in healthy women with obesity.

## Materials and methods

### Study design and participants

In this double-blind, controlled, randomized clinical trial (RCT), implemented between February and August 2019 at the Nutrition Research Center, Tabriz University of Medical Sciences (TBZMED, Tabriz, Iran), 46 eligible volunteer women with obesity, unwilling to follow weight-reducing diets, aged 19–49 years, and body mass index (BMI) of 30–35 kg/m^2^ were recruited through announcement and prescreened for enrollment via phone interview.

The exclusion criteria were as follows: pregnancy, lactation, menopause; history of diabetes, hypertension, cardiovascular, thyroid, renal, gastrointestinal, hepatic, or active infectious diseases; physical disability; intestinal surgeries, vegetarianism or veganism; vigorous physical activity; following weight loss diets, taking weight-reducing supplements/medications, pro/pre/synbiotics, multivitamins, antacids, antibiotics, or laxative medicines within the last 2 months; taking drugs influencing glucose/lipid metabolism, i.e., antidiabetics, lipid-lowering agents, glucocorticoids, contraceptives, non-steroidal anti-inflammatory drugs (NSAIDs), β-blockers; steroids, immunosuppressive or anticonvulsant medicines; and smoking or alcohol consumption. At baseline, all participants were requested to sign a written informed consent after a full explanation of the research process; afterward, demographic, medical history, and physical activity questionnaires were filled out for them. Subsequently, anthropometric measurements and the 24-h recall interview were conducted by a nutritionist.

This research was performed in accordance with the Declaration of Helsinki principles, approved by the Ethics Committee of the TBZMED (ethics-code: IR.TBZMED.REC.1396.747), and registered with the Iranian-Registry-of-Clinical-Trials (http://www.irct.ir, id: IRCT20080904001197N13).

### Sample size

Considering 95% confidence level and 80% power in two-tailed tests, based on changes in weight obtained from a previous pilot clinical trial reporting a significant decrease in body weight (82.0 ± 2.2 vs. 80.9 ± 1.8 kg, *p* = 0.007) after carnitine supplementation for 4 weeks (44), a minimum sample size of 17 was determined for each group, which was increased to 23, anticipating a dropout rate of 35%, using the Power Analysis and Sample Size Software (PASS; NCSS, LLC, USA).

### Randomization, blinding, and intervention

Following a 2-week run-in period, the participants were randomly allocated to the “L-carnitine + synbiotic” or “L-carnitine + placebo” groups via block randomization in a 1:1 ratio, in blocks of two, stratified by age, using the Random Allocation Software (RAS). Intervention allocation was blinded for the participants and researchers; to assure concealment, participants’ allocation was performed via consecutively numbered, opaque, sealed envelopes, by an investigator not involved in the study. The L-carnitine + synbiotic intervention group received two L-carnitine tablets/day (500 mg of L-carnitine tartrate/tablet, Karen Company, Iran) after the main meals, plus multistrain/multispecies synbiotic [Probiotics International Ltd. (Protexin^®^), Lopen Head, Somerset, UK] and one 250 mg capsule/day after lunch containing 175 mg fructo-oligosaccharide (FOS), plus 1 × 10^8^ colony-forming unit (CFU)/capsule, freeze-dried Protexin probiotics, including *Bifidobacterium breve PXN 25*, *Bifidobacterium longum PXN 30*, *Lactobacillus casei PXN 37*, *Lactobacillus rhamnosus PXN 54*, *Lactobacillus acidophilus PXN 35*, *Lactobacillus bulgaricus PXN 39*, and *Streptococcus thermophilus PXN 66*, while those in the L-carnitine + placebo group received the same amounts of L-carnitine and 250 mg maltodextrin capsule/day, as the synbiotic placebo (FIC Co., China), for eight sequential weeks. Furthermore, all participants received healthy-eating dietary advice according to the Food Guide Pyramid and the National Heart, Lung, and Blood Institute (NHLBI) Obesity Education Initiative Expert Panel guideline by a nutritionist (45).

Participants received the supplements/placebo in monthly visits. Placebo and synbiotic capsules were identical in color, shape, and size. To ensure adherence and discuss possible adverse effects, subjects received twice weekly phone calls and were asked to mention the side effects and to return the unused tablets/capsules. Compliance, described as taking at least 90% of the supplements, was assessed by the returned pill count (46), through which the returned tablets/capsules were counted. Moreover, participants were asked not to change routine dietary and physical activity habits during the study to eliminate the probable confounding impacts of dietary/physical activity alterations on the study results, as well as not to use pro/pre/synbiotic-supplemented food products during the intervention period.

### Physical activity, dietary intake, and anthropometric assessments

Physical activity level (PAL) and dietary intake were evaluated at three time points: weeks 1 (baseline), 4, and 8. Anthropometric measurements, including height, BMI, body weight, and neck, waist, and hip circumferences (NC, WC, HC), were performed pre- and post-intervention. Height and weight were measured to the nearest 0.1 cm and 0.1 kg in light clothing without shoes by a calibrated stadiometer and scale (Seca, Hamburg, Germany). BMI was calculated as weight/height^2^ (kg/m^2^). NC was measured at mid-neck height, between the mid-cervical spine and mid-anterior neck while sitting with a straight back, all to the nearest 0.1 cm; WC at the narrowest horizontal girth between the costal and iliac crests (47); and HC at the widest circumference over the greater trochanters, using a flexible inelastic measuring tape. Waist/hip ratio (WHR) was calculated as WC/HC. Dietary intake was evaluated via three 24-h dietary recalls (two non-sequential days and a weekend), analyzed using the Nutritionist IV software, modified for Iranian foods (First Databank Inc, San Bruno, CA, USA). The International Physical Activity Questionnaire-Short Form (IPAQ-SF) was applied to assess PAL through in-person interviews (48). Based on the IPAQ analysis guidelines, the metabolic equivalent of tasks score (MET—min/week) was calculated, classifying the participants as low (<600 MET), moderate (600–3,000 MET), or severe active (≥3,000 MET) (48). A nutritionist conducted the stated assessments.

### Biochemical assays

Following a 12-h overnight fasting, venous blood (5 ml) was obtained for biochemical analyses, pre- and post-intervention. Serum TC, TG, HDL-C, FBS, and insulin were measured by colorimetric enzymatic methods, using commercial kits (Pars-Azmoon Co, Tehran, Iran); low-density lipoprotein-cholesterol (LDL-C) was calculated by the Friedewald formula. Insulin level was determined using the enzyme-linked immunosorbent assay (ELISA) kit (Monobind, Lake Forest, CA, USA). Appropriate formulas were used to calculate QUICKI and HOMA-IR (49, 50).

### Statistical analysis

Statistical analysis was carried out using SPSS Release 23.0 software (SPSS Inc., Chicago, IL, USA). The Kolmogorov–Smirnov test was applied to assess the normality of quantitative variables distribution. Frequency (percentage) was presented for categorical data and mean (SD) for normally distributed numerical variables. Data analysis was conducted based on both per-protocol and intention-to-treat (ITT) approaches (51), using the multiple imputation procedure for missing values imputation as for the ITT approach. Moreover, participants with an adherence rate of less than 90% or those unwilling to continue the study were planned to be excluded, concerning the per-protocol method. To compare between-group baseline disparities, the independent samples *t*-test was used for numerical variables, while the Pearson chi-square or the trend chi-square test was used for categorical variables. To assess within-group changes, paired-samples *t*-test or repeated-measures analysis of variance was applied. The trend chi-square test was applied to determine intragroup differences of qualitative variables. To avoid potential bias in assessing intergroup post-intervention differences, the analysis of covariance (ANCOVA) test was applied while adjusting for possible confounding factors, such as baseline values, age, changes in energy intake, physical activity, and BMI. The relative effect size was expressed as percent of changes (PC), calculated as follows: [(post-intervention value − baseline value)/baseline value) × 100)]. To assess the clinical effectiveness of the intervention, the number needed to treat (NNT), an absolute measure of effectiveness, was calculated as the inverse of absolute risk reduction (ARR) as an estimate of the overall clinical impact (NNT = 1/ARR), considering the median of weight reduction in the studied population (≥2.45 kg) as optimal. Statistical significance was defined as *p*-value <0.05.

## Results

### General characteristics of the study

**Figure 1:**
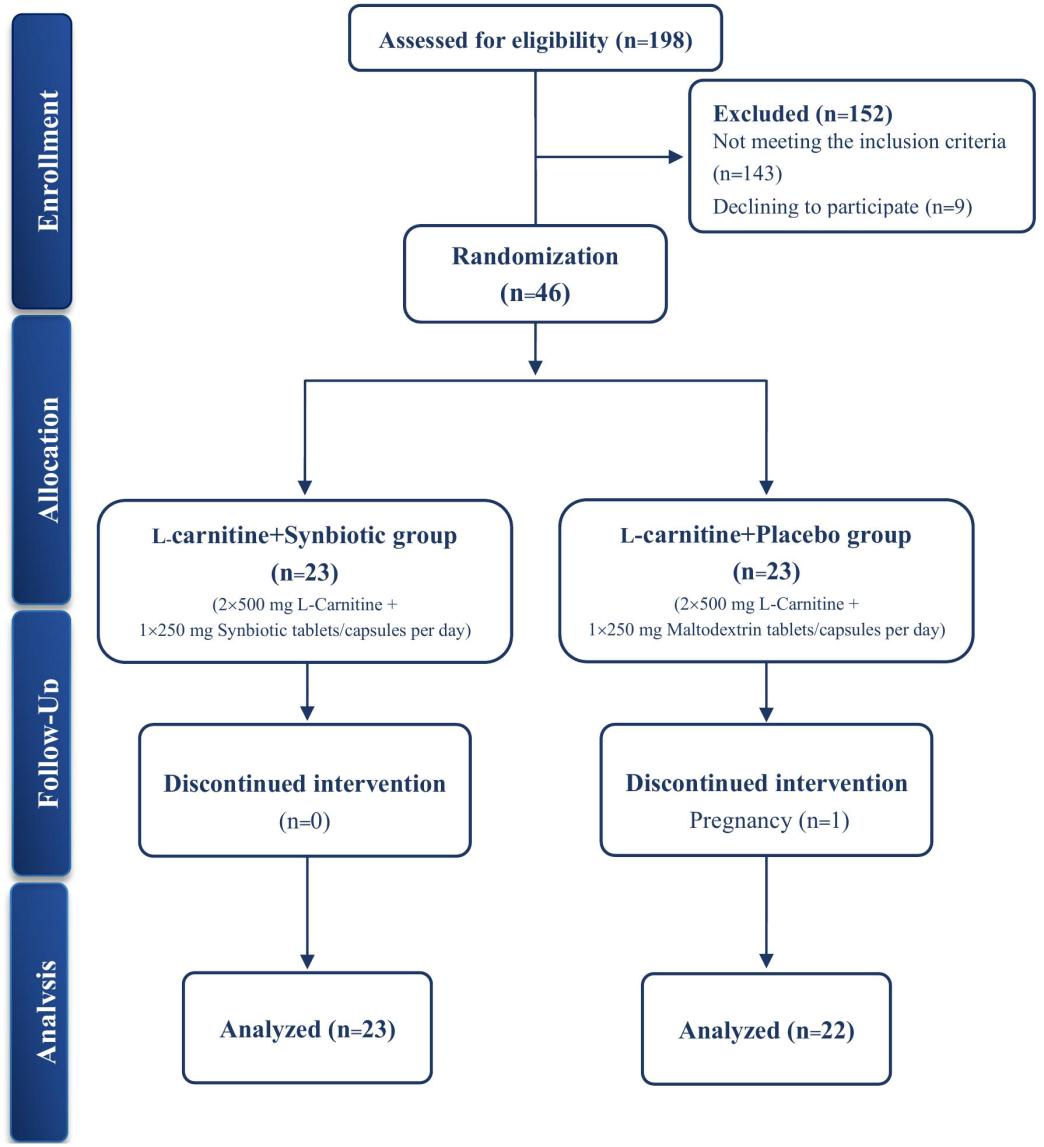
The study flow diagram.

In total, of the 198 screened volunteers, 46 qualified participants were recruited, among which 45 completed the study with one participant in the placebo group discontinuing the study because of pregnancy (**Figure 1**). Data analysis performed based on both per-protocol (*n* = 45) and intention-to-treat (ITT) (*n* = 46) statistical approaches showed comparable results (the complete set of ITT analysis results are presented as **Supplementary Material**). No side effects were stated, except for temporary, mild gastrointestinal symptoms in one participant in the L-carnitine + synbiotic group. According to the returned pill count method, mean compliance rates were 93.93% and 94.40% for the L-carnitine + placebo group vs. the L-carnitine + synbiotic group, respectively (46). No significant intergroup differences were observed in the baseline characteristics or PAL (**Table 1**).

**Table 1:** Baseline characteristics of the participants.

Variable	L-carnitine + synbiotic (*n* = 23)	L-carnitine + placebo (*n* = 22)	*p*
**Age (years)**	38.39 (6.30)	38.00 (7.47)	0.850^a^
**Weight (kg)**	84.04 (8.67)	85.01 (7.88)	0.697^a^
**Height (cm)**	160.56 (6.02)	159.95 (6.79)	0.751^a^
**BMI (kg/m^2^)**	32.59 (2.02)	33.22 (1.75)	0.273^a^
**PAL (MET—min/week)**	776.95 (276.83)	733.09 (294.83)	0.609^a^
**Marital status, *n* (%)** Single Married Divorced or widow	3 (13.00) 19 (82.6) 1 (4.3)	4 (18.2) 18 (81.8) 0 (0.0)	0.483^b^
**Education, *n* (%)** Illiterate Up to high school University degree	0 (0.0) 11 (47.8) 12 (52.2)	1 (4.5) 12 (54.5) 9 (40.9)	0.414^b^
**Occupation, *n* (%)** Housewife Employee Other	11 (47.8) 11 (47.8) 1 (4.4)	16 (72.7) 6 (27.3) 0 (0.0)	0.098^b^

Numerical data are expressed as mean (SD) and categorical variables as number (%).

a p-value based on the independent samples t-test.

b p-value based on the chi-square test.

### Physical activity, dietary intake, and anthropometric measurements

**Figure 2:**
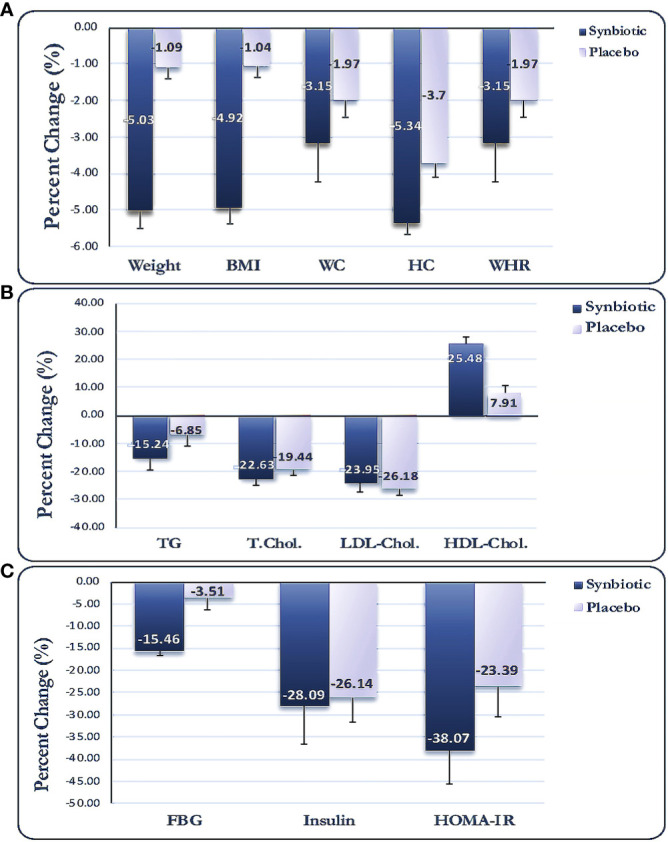
Percent changes for anthropometric **(A)**, lipid profile **(B)**, and glycemic indices **(C)**. Data expressed as mean (SD). BMI, body mass index; WC, waist circumference; HC, hip circumference; WHR, waist to hip ratio; TG, triglyceride; TC, total cholesterol; LDL-C, low-density lipoprotein-cholesterol; HDL-C, high-density lipoprotein-cholesterol; FBS, fasting blood sugar; HOMA-IR, homeostasis model assessment of insulin resistance.

No significant disparities were observed in the physical activity (METs) or dietary intake at baseline in either group (**Table 2**); moreover, no significant within-/between-group disparities were noted for the stated factors, even after adjustment for baseline values or other potential confounders. Anthropometric indices were not significantly different between the two study groups at baseline (**Table 3**); nevertheless, BMI, body weight, NC, WC, HC, and WHR reduced significantly in both groups after the intervention. Additionally, the L-carnitine + synbiotic group indicated a larger decrease in the above-stated parameters, except for WHR, compared with the other group (*p* < 0.001, <0.001, <0.001, = 0.012, and =0.030, respectively), even after adjustment for the baseline levels, changes in energy intake, and physical activity as the potential confounders (**Table 3**). The percent of changes (PCs) for weight were −5.03% and −1.09% in the L-carnitine + synbiotic and L-carnitine + placebo groups, respectively (**Figure 2A**).

**Table 2:** Dietary intake and physical activity of the participants throughout the study.

Variable	L-carnitine + synbiotic (*n* = 23)	L-carnitine + placebo (*n* = 22)	MD (95% CI) (between groups)	*p*
Energy (kcal)				
Baseline After 4 weeks After 8 weeks *p* ^a^	2,390.21 (660.67) 2,322.78 (590.99) 2,300.91 (541.14) 0.161	2,086.31 (461.49) 1,974.22 (456.78) 1,950.72 (385.79) 0.188	303.89 (−40.18, 647.98) 75.26 (−24.10, 174.62)^f^ 144.72 (−39.33, 328.79)^f^	0.082^b^ 0.134^c^ 0.120^c^
Protein (g)				
Baseline After 4 weeks After 8 weeks *p* ^a^	72.41 (17.43) 69.66 (13.59) 73.47 (14.91) 0.377	69.35 (20.00) 62.76 (13.86) 64.23 (22.33) 0.228	3.06 (−8.20, 14.33) 4.67 (−1.88, 11.24)^f^ 8.06 (−1.05, 17.17)^f^	0.586^b^ 0.158^d^ 0.081^d^
Carbohydrate (g)				
Baseline After 4 weeks After 8 weeks *p* ^a^	313.25 (104.96) 302.84 (94.28) 298.59 (117.49) 0.249	263.47 (80.94) 243.93 (64.86) 250.08 (68.13) 0.150	49.78 (−6.75, 106.31) 10.34 (−10.21, 30.91)^f^ 1.23 (−28.51, 30.98)^f^	0.083^b^ 0.315^d^ 0.934^d^
Fat (g)				
Baseline After 4 weeks After 8 weeks *p* ^a^	90.04 (24.25) 87.48 (18.11) 88.21 (23.24) 0.694	88.16 (27.71) 81.15 (18.37) 77.89 (24.30) 0.211	1.88 (−13.75, 17.52) 4.90 (−0.96, 10.77)^f^ 7.44 (−2.28, 17.17)^f^	0.809^b^ 0.099^d^ 0.130^d^
Dietary fiber (g)				
Baseline After 4 weeks After 8 weeks *p* ^a^	11.66 (4.74) 11.85 (4.60) 10.83 (4.74) 0.268	10.12 (4.08) 11.66 (2.63) 10.90 (5.48) 0.264	1.53 (−1.12, 4.20) −0.82 (−2.53, 0.88)^f^ −1.24 (−3.93, 1.43)^f^	0.251^b^ 0.334^d^ 0.353^d^
Physical activity (METs)				
Baseline After 4 weeks After 8 weeks *p* ^a^	776.95 (276.83) 803.47 (256.76) 784.56 (282.65) 0.230	733.09 (294.83) 732.27 (322.86) 734.36 (311.91) 0.970	43.86 (−127.99, 215.73) 27.40 (−7.26, 62.07)^f^ 5.70 (−34.61, 46.02)^f^	0.609^b^ 0.118^e^ 0.777^e^

Data are presented as mean (SD) and mean difference (95% CI).

METs, metabolic equivalents (MET—min/week); MD, mean difference; CI, confidence interval.

a p-value based on repeated-measures analysis of variance (RM-ANOVA) (comparison of data with more than two measurements within the groups post-intervention).

b p-value based on the independent samples t-test (comparison of data between the groups at baseline).

c p-value based on analysis of covariance (ANCOVA) (comparison of data between the groups post-intervention, adjusted for baseline values and changes in physical activity).

d p-value based on analysis of covariance (ANCOVA) (comparison of data between the groups post-intervention, adjusted for baseline values, changes in physical activity, and energy intake).

e p-value based on ANCOVA (comparison of data between the groups post-intervention, adjusted for baseline values).

f Absolute effect size (95% CI) based on the mentioned ANCOVA models; bold values indicate statistically significant differences (p < 0.05).

**Table 3:** Changes in anthropometric indices of the participants throughout the study.

Variable	L-carnitine + synbiotic (*n* = 23)	L-carnitine + placebo (*n* = 22)	MD (95% CI) (between groups)	*P*
Weight (kg)				
Baseline After 8 weeks **MD (95% CI) (within groups)** *p* ^a^	84.04 (8.67) 79.81 (7.42) −4.23 (−5.18, −3.26) **<0.001**	85.01 (7.88) 84.09 (7.46) −0.92 (−1.56, −0.27) **0.007**	−0.97 (−5.96, 4.02) −3.41 (−4.35, −2.47)^d^	0.697^b^ **<0.001** ^c^
BMI (kg/m^2^)				
Baseline After 8 weeks **MD (95% CI) (within groups)** *p* ^a^	32.59 (2.02) 30.98 (1.88) −1.61 (−1.94, −1.28) **<0.001**	33.22 (1.75) 32.87 (1.59) −0.35 (−0.60, −0.11) **0.007**	−0.63 (−1.76, 0.51) −1.33 (−1.70, −0.95)^d^	0.273^b^ **<0.001** ^c^
WC (cm)				
Baseline After 8 weeks **MD (95% CI) (within groups)** *p* ^a^	101.39 (8.82) 92.97 (7.37) −8.41 (−10.87, −5.94) **<0.001**	103.95 (8.02) 97.86 (6.47) −6.09 (−7.45, −4.73) **<0.001**	−2.56 (−7.63, 2.51) −3.04 (−5.38, −0.70)^d^	0.314^b^ **0.012** ^c^
HC (cm)				
Baseline After 8 weeks **MD (95% CI) (within groups)** *p* ^a^	116.30 (5.77) 110.04 (4.70) −6.26 (−7.14, −5.37) **<0.001**	117.54 (6.03) 112.65 (5.52) −4.88 (−6.47, −3.29) **<0.001**	−1.24 (−4.79, 2.30) −1.70 (−3.23, −0.17)^d^	0.485^b^ **0.030** ^c^
WHR				
Baseline After 8 weeks **MD (95% CI) (within groups)** *p* ^a^	0.87 (0.06) 0.84 (0.06) −0.02 (−0.04, −0.009) **0.006**	0.88 (0.06) 0.87 (0.06) −0.01 (−0.02, −0.003) **0.014**	−0.01 (−0.05, 0.02) −0.01 (−0.03, 0.007)^d^	0.489^b^ 0.165^c^
NC				
Baseline After 8 weeks **MD (95% CI) (within groups)** *p* ^a^	38.33 (1.62) 36.75 (1.57) −1.58 (−1.88, −1.28) **<0.001**	38.81 (1.28) 38.43 (1.07) −0.38 (−0.62, −0.13) **0.004**	−0.47 (−1.35, 0.40) −1.27 (−1.63, −0.91)^d^	0.283^b^ **<0.001** ^c^

Data are presented as mean (SD) and mean difference (95% CI).

BMI, body mass index; WC, waist circumference; HC, hip circumference; WHR, waist to hip ratio; NC, neck circumference; MD, mean difference; CI, confidence interval.

a p-value based on the paired samples t-test (comparison of data within the groups post-intervention).

b p-value based on the independent samples t-test (comparison of data between the groups at the baseline).

c p-value based on analysis of covariance (ANCOVA) (comparison of data between the groups post-intervention, adjusted for baseline values, changes in physical activity, and energy intake).

d Absolute effect size (95% CI) based on the mentioned ANCOVA models; bold values indicate statistically significant differences (p < 0.05).

### Biochemical factors

Alterations in biochemical indices are shown in **Table 4**. No significant differences were observed for the lipid or glycemic indices between the two groups at baseline. Serum concentrations of TG, TC, and LDL-C decreased, while HDL-C increased significantly in the L-carnitine + synbiotic group compared with the baseline (*p* < 0.001 for all), and the lipid profile changed significantly in the L-carnitine + placebo group post-intervention (*p* < 0.001, <0.001, = 0.030, respectively), except for TG (*p* = 0.077). Moreover, there were no significant intergroup disparities for neither of the lipid indicators post-intervention, even when adjusted for the baseline levels and probable confounders, except for HDL-C (*p* = 0.009). Moreover, a significant reduction in glycemic indices (FBS, insulin, HOMA-IR, *p* < 0.001) and an increase in QUICKI (*p* < 0.001) were noted in the L-carnitine + synbiotic group in comparison to the baseline, except for FBS (*p* = 0.151), and alterations in glycemic indices were significant in the L-carnitine + placebo group (*p* = 0.026, 0.016, 0.023, respectively). Additionally, a profound decline in FBS, insulin, and HOMA-IR (*p* = 0.014, 0.051, 0.024, respectively) and an increment in QUICKI (*p* = 0.019) were observed in the L-carnitine + synbiotic group compared with the L-carnitine + placebo group post-intervention, which remained significant after adjustment for the baseline levels and possible confounding factors. PCs for the metabolic parameters are presented in **Figures 2B, C**.

**Table 4:** Changes in lipid profile and glycemic indices of the participants throughout the study.

Variable	L-carnitine + synbiotic (*n* = 23)	L-carnitine + placebo (*n* = 22)	MD (95% CI) (between groups)	*p*
TG (mg/dl)				
Baseline After 8 weeks **MD (95% CI) (within groups)** *p* ^a^	124.73 (39.21) 101.30 (26.26) −23.43 (−35.01, −11.85) **<0.001**	111.54 (35.87) 101.81 (28.79) −9.72 (−20.61, 1.16) 0.077	13.19 (−9.43, 35.81) −7.82 (−24.20, 8.56)^d^	0.246^b^ 0.340^c^
TC (mg/dl)				
Baseline After 8 weeks **MD (95% CI) (within groups)** *p* ^a^	189.34 (25.38) 145.47 (18.08) −43.86 (−53.62, −34.11) **<0.001**	191.50 (39.18) 153.86 (28.18) −37.63 (−49.76, −25.50) **<0.001**	−2.15 (−22.21, 17.90) −5.17 (−21.11, 10.77)^d^	0.829^b^ 0.516^c^
LDL-C (mg/dl)				
Baseline After 8 weeks **MD (95% CI) (within groups)** *p* ^a^	112.17 (22.98) 84.08 (15.27) −28.08 (−36.61, −19.55) **<0.001**	125.54 (31.50) 92.63 (24.66) −32.90 (−43.03, -22.78) **<0.001**	−13.37 (−29.89, 3.15) −0.39 (−14.73, 13.94)^d^	0.110^b^ 0.956^c^
HDL-C (mg/dl)				
Baseline After 8 weeks **MD (95% CI) (within groups)** *p* ^a^	41.08 (8.65) 51.69 (11.10) 10.60 (8.19, 13.01) **<0.001**	40.86 (7.15) 43.72 (7.42) 2.86 (0.30, 5.42) **0.030**	0.22 (−4.56, 5.01) 6.75 (1.75, 11.74)^d^	0.925^b^ **0.009** ^c^
FBS (mg/dl)				
Baseline After 8 weeks **MD (95% CI) (within groups)** *p* ^a^	89.95 (8.88) 75.65 (4.75) −14.30 (−17.06, −11.54) **< 0.001**	85.81 (11.54) 81.72 (8.67) −4.09 (−9.80, 1.62) 0.151	4.13 (−2.04, 10.31) −7.57 (−13.54, −1.60)^d^	0.184^b^ **0.014** ^c^
Insulin (µIU/ml)				
Baseline After 8 weeks **MD (95% CI) (within groups)** *p* ^a^	19.83 (12.69) 10.50 (3.19) −9.32 (−14.46, −4.18) **<0.001**	20.00 (9.88) 14.34 (5.28) −5.66 (−10.56, −0.75) **0.026**	−0.17 (−7.03, 6.69) −3.56 (−7.15, 0.01)^d^	0.960^b^ 0.051^c^
HOMA-IR				
Baseline After 8 weeks **MD (95% CI) (within groups)** *p* ^a^	4.37 (2.79) 1.97 (0.66) −2.40 (−3.52, −1.27) **<0.001**	4.38 (2.47) 2.93 (1.17) −1.44 (−2.59, −0.29) **0.016**	−0.007 (−1.59, 1.58) −0.89 (−1.66, −1.28)^d^	0.993^b^ **0.024** ^c^
QUICKI				
Baseline After 8 weeks **MD (95% CI) (within groups)** *p* ^a^	0.31 (0.03) 0.34 (0.01) 0.02 (0.01, 0.04) **<0.001**	0.31 (0.02) 0.32 (0.01) 0.01 (0.001, 0.023) **0.023**	0.003 (−0.01, 0.02) 0.01 (0.003, 0.031)^d^	0.725^b^ **0.019** ^c^

Data are presented as mean (SD) and mean difference (95% CI).

TG, triglyceride; TC, total cholesterol; LDL-C, low-density lipoprotein-cholesterol; HDL-C, high-density lipoprotein-cholesterol; FBS, fasting blood sugar; HOMA-IR, homeostasis model assessment of insulin resistance; QUICKI, quantitative insulin sensitivity check index; MD, mean difference; CI, confidence interval.

a p-value based on the paired samples t-test (comparison of data within the groups post-intervention).

b p-value based on the independent samples t-test (comparison of data between the groups at baseline).

c p-value based on analysis of covariance (ANCOVA) (comparison of data between the groups post-intervention, adjusted for baseline values, changes in physical activity, energy intake, and BMI).

d Absolute effect size (95% CI) based on the mentioned ANCOVA models; bold values indicate statistically significant differences (p < 0.05).

### Clinical effectiveness of intervention

The clinical effectiveness of the intervention on body weight is presented in **Table 5**. Out of 23 participants in the L-carnitine + synbiotic group, 19 (82.6%) experienced optimal amelioration in body weight post-intervention, defined as the median of weight reduction in the studied population (≥2.45 kg), while only 5 of the 22 participants (22.7%) in the L-carnitine + placebo group experienced the expected weight loss. ARR (95% CI) for the L-carnitine + synbiotic vs. the L-carnitine + placebo group was 59.8% (0.36–0.83), and the NNT to achieve the defined weight loss throughout the 8-week intervention was calculated as 2, which indicates that approximately one in every two participants in the L-carnitine + synbiotic group will benefit from intervention.

**Table 5:** Clinical effectiveness of the intervention for body weight reduction.

Optimal reduction in body weight	Intervention group	Number of participants with improvement, *n* (%)	ARR (95% CI)	NNT
≥2.45 (kg)	L-carnitine + synbiotic (*n* = 23)	19 (82.6%)	0.59 (0.036–0.83)	2
	L-carnitine + placebo (*n* = 22)	5 (22.7%)		

Median of weight loss in the studied population (≥2.45 kg) was defined as optimal weight reduction.

ARR, absolute risk reduction; CI, confidence interval; NNT, number needed to treat.

## Discussion

**Figure 3:**
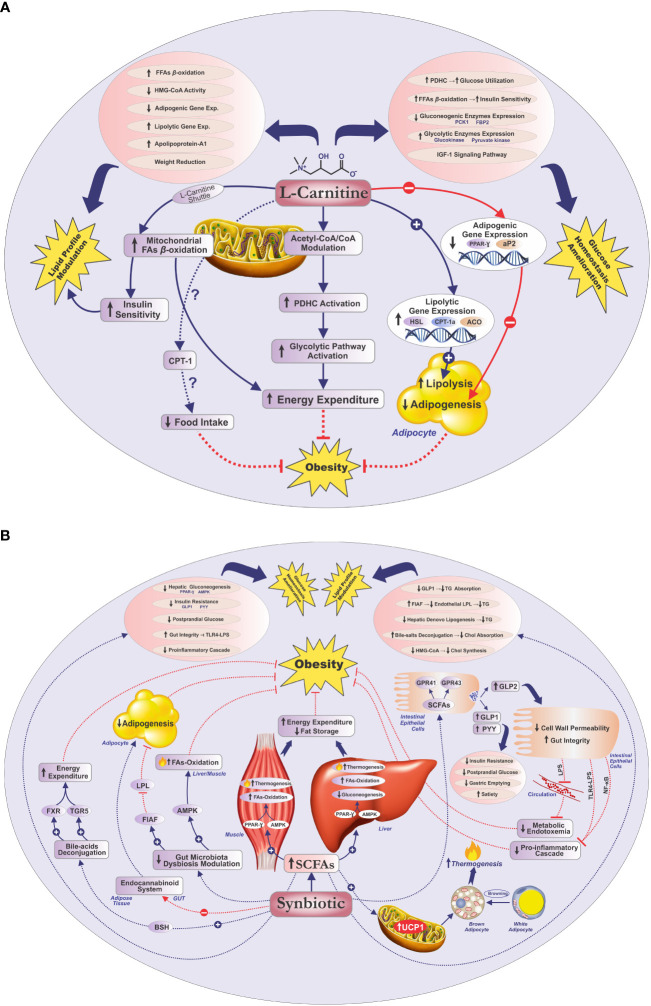
Potential mechanisms for the improving effects of L-carnitine/synbiotics on obesity, lipid profile, and glycemic indices **(A, B)**. Acetyl-CoA, acetyl coenzyme-A; ACO, acyl-coenzyme-A oxidase; AMPK, adenosine monophosphate-activated protein kinase; aP2, adipose-specific fatty acid-binding protein; BSH, bile salt hydrolase; Chol, cholesterol; CO-A, coenzyme A; CPT-I(a), carnitine palmitoyltransferase I(a); Exp, expression; FAs, fatty acids; FBP2, fructose-1, 6-bisphosphatase isozyme 2; FFAs, free fatty acids; FIAF, fasting-induced adipose factor; FXR, farnesoid X receptor; GPR41, G protein-coupled receptor 41; GPR43, G protein-coupled receptor 43; GLP-1, glucagon-like peptide-1; GLP-2, glucagon-like peptide 2; HSL, hormone-sensitive lipase; HMG-CoA, hydroxymethylglutaryl CoA reductase; IGF-1, insulin-like growth factor 1; LPL, lipoprotein lipase; LPS, lipopolysaccharide; NF-κB, nuclear factor kappa B; PCK1, phosphoenolpyruvate carboxykinase; PDHC, pyruvate dehydrogenase complex; PPAR-γ, peroxisome proliferator-activated receptor-gamma; PYY, peptide YY; SCFAs, short-chain fatty acids; TG, triglyceride; TGR5, Takeda G-protein-coupled bile acid receptor 5; TLR4-LPS, Toll-like receptor 4-LPS; UCP-1, uncoupling protein-1.

The present study evaluated the impacts of co-supplementing L-carnitine with multistrain/multispecies synbiotic vs. L-carnitine concomitant with placebo on anthropometric and metabolic profiles in women with obesity. Both “L-carnitine + synbiotic” and “L-carnitine + placebo” supplementation significantly ameliorated the anthropometric indices, lipid profile, and glucose homeostasis parameters, except for the reduction of TG and FBS in the L-carnitine + placebo group. Importantly, L-carnitine + synbiotic co-supplementation significantly alleviated the anthropometric and glycemic indices, compared with the L-carnitine + placebo; nonetheless, no significant intergroup disparities were detected for the lipid indices, except for HDL-C. Overall, the intragroup findings of this study indicate that both L-carnitine + synbiotic and L-carnitine + placebo supplementation might have beneficial effects on obesity and related cardiometabolic biomarkers. Nevertheless, according to the statistically significant intergroup disparities revealed for the anthropometric (BMI, weight, NC, WC, and HC) and glycemic indices (FBS, insulin, HOMA-IR, QUICKI) and HDL-C, co-supplementation of L-carnitine with synbiotic was more advantageous to improve these parameters. Based on our in-depth search, there were no reports on the L-carnitine + synbiotic joint supplementation effects on the stated factors in individuals with obesity; therefore, we discussed our findings based on the literature indicating the impacts of either supplement separately. The conceivable mechanisms for the ameliorating impacts of either synbiotics or L-carnitine on obesity and glycemic/lipid indices are illustrated in **Figures 3A, B**.

Although preclinical investigations have implied the efficiency of L-carnitine in weight reduction (52), clinical evidence regarding its anti-obesity effects is contradictory. The present study demonstrated that L-carnitine + synbiotic joint administration significantly reduced BMI, body weight, NC, WC, and HC compared with L-carnitine + placebo. Additionally, post-intervention intragroup differences for these variables were also significant in both groups. Our findings regarding the ameliorating impacts of L-carnitine on anthropometric measures were in line with the findings of recent meta-analyses, which concluded that L-carnitine prescription could amend anthropometric measures (11, 12).

Multiple plausible mechanisms are suggested for the beneficial effects of carnitine on increasing energy expenditure and subsequent weight loss. Carnitine plays a pivotal role in fatty acid ß-oxidation, through facilitating the translocation of long-chain fatty acids across the mitochondrial membrane (8, 18). Moreover, it may increase energy expenditure by modulating the mitochondrial acetyl-CoA/CoA ratio (19), thus activating the pyruvate dehydrogenase complex (PDHC) (8, 19), leading to the activation of the glycolytic pathway (8, 10). In addition, L-carnitine might protect against the decrease in metabolic rate during the weight reduction period through enhancing the resting energy expenditure (REE) (52). Furthermore, the anti-obesity properties of carnitine might be attributed to its potential role in stimulating adipocyte lipolysis, through upregulating lipolytic gene expression, namely, hormone-sensitive lipase (HSL) (21), carnitine palmitoyl transferase Ia (CPT-Ia) (21), and acyl coenzyme A oxidase (ACO) (21), as well as attenuating adipogenesis in adipocytes through suppression of adipogenic gene expression, including peroxisome proliferator-activated receptor gamma (PPAR-γ) and adipose-specific fatty acid-binding protein (aP2) in adipose tissue (21). Finally, it has been reported that modulation of CPT-1 might have an improving effect on food intake and energy metabolism (20).

According to the evidence demonstrating altered gut microbiota diversity or composition in obesity (53), remodeling the gut microbiota through pro/pre/synbiotic supplementation is speculated as a promising preventive/therapeutic strategy in obesity (24, 25). It has been suggested that multistrain probiotic/synbiotic therapy might induce complementary or synergistic effects in metabolic disorders (38), compared with single-strain probiotics or pre/probiotics monotherapy (25). Our findings regarding anthropometric indices were consistent with the findings of a meta-analysis, indicating that multistrain probiotic administration markedly diminished body weight and BMI (54). Moreover, a recent meta-analysis illustrated a significant reduction in weight and WC due to synbiotic supplementation (55). Notably, in a recent umbrella review of 14 meta-analyses, assessing pro/synbiotics on weight changes, most studies revealed a decrease in BMI and/or weight, favoring pro/synbiotics compared with placebo (26).

Several possible mechanisms have been conceived for the favorable impacts of synbiotics supplementation on anthropometric indices, mediated through the complementary/synergistic effects of their pro- and prebiotic compartments (24, 38). Collectively, the weight-reducing properties of synbiotics could be ascribed to their possible contribution in modulating energy homeostasis, elevating anorexigenic hormones (30), blunting appetite (30), and alleviating systemic inflammation (56, 57). Accordingly, the probable mechanisms could be discussed in four main approaches, namely, modulation of gut microbiota dysbiosis, production of SCFAs, reduction of gut permeability and metabolic endotoxemia, and regulation of bile acid metabolism (32, 35, 36).

Evidence has intimated that synbiotics might reverse the detrimental effects of microbiota dysbiosis, promoting weight loss and maintenance via restoring healthy gut microbiota composition and/or function (35, 36). According to literature, gut microbiota modulation might lead to a decline in fat storage in adipocytes due to incrementing fasting-induced adipose factor (FIAF) levels, a suppressor of lipoprotein lipase (LPL) (30), and foster lipid oxidation in the liver and muscles via amplified activity of adenosine monophosphate-activated protein kinase (AMPK) (30).

Growing evidence has depicted that gut microbiota modification leads to augmented SCFA production, which underlies most of the aforementioned beneficial impacts (32). It has been indicated that SCFAs may improve energy homeostasis and fat storage via boosting fatty acid oxidation and thermogenesis through upregulating the expression of peroxisome proliferator-activated receptor gamma coactivator-1α (PGC-1α) and AMPK in the muscle and liver tissues. Moreover, SCFAs might induce thermogenesis in brown adipose tissue (BAT), through upregulating mitochondrial uncoupling protein-1 (UCP-1), and white adipose tissue (WAT) browning (37, 58). Furthermore, SCFAs might regulate energy homeostasis through binding to the G protein-coupled receptors (GPCRs), GPR41 and GPR43, on intestinal epithelial cells (53), resulting in enhanced production and secretion of gut hormones (25, 32, 56), namely, peptide YY (PYY) (59) and glucagon-like peptides 1 and 2 (GLP-1 and GLP-2) (60). PYY and GLP-1 might delay gastric emptying (61), enhance satiety (25, 32, 62), and decrease fat mass (62), whereas GLP-2 contributes to the reduced permeability of the intestinal wall and metabolic endotoxemia (63) due to diminished entrance of lipopolysaccharide (LPS), a gram-negative bacteria cell wall component, into systemic circulation (63). Furthermore, probiotics might be involved in bile acid metabolism, via producing bile salt hydrolase (BSH) (64), through activating farnesoid X receptor (FXR) and Takeda G-protein-coupled bile acid receptor 5 (TGR5), thereby regulating energy expenditure (65). Finally, prebiotics might be implicated in diminishing adipogenesis, via inhibiting the endocannabinoid system in the gut and adipose tissue (60, 66).

Regarding the lipid profile, despite the significant intragroup amelioration in lipid indices, except for TG in the L-carnitine + placebo group, no significant disparities were found for the lipid parameters among the groups, except for HDL-C. Our failure to find significant intergroup differences in lipid profile could be due to the fact that both carnitine and synbiotics might induce beneficial effects on lipid profile (15, 28); consequently, both groups experienced a comparable decline in lipid parameters, which resulted in the insignificant intergroup differences. Furthermore, this might probably be the consequence of the within-normal range of primary levels of these indicators, a rather short period of intervention, or possibly the inadequate dose of synbiotics to convince more advantageous modifications in the joint-supplemented group. Meanwhile, the significant intergroup disparity for HDL-C could be ascribed to the possible complementary/synergetic properties of synbiotic and L-carnitine compared with L-carnitine alone or possibly due to the greater alterations in anthropometric indices in the joint-supplemented group (67). Finally, the significant intragroup reduction of TG in the L-carnitine + synbiotic group (*p* < 0.001), which was not found in the other group (*p* = 0.077), might be rationalized by the complementary/synergistic effects of synbiotic and L-carnitine in declining TG levels or could have been intervened via the greater weight reduction in the L-carnitine + synbiotic group (68).

Our results regarding the hypolipidemic properties of L-carnitine were in accordance with the findings of recent meta-analyses, addressing the ameliorating effects of L-carnitine on TG, TC, LDL-C, and HDL-C (14, 15). The suggested mechanisms for the beneficial impacts of L-carnitine on lipid profile include reducing the conversion of free fatty acids (FFAs) to triglycerides (14); decreasing insulin resistance (69); stimulating apolipoprotein-A1 production, which is the major apolipoprotein of HDL-C (14); and blunting cholesterol synthesis via prohibiting the hydroxymethylglutaryl CoA reductase (HMG-CoA) activity (70), being implicated in weight reduction and consequently lipid profile improvement (68). Moreover, alleviating adipogenesis via downregulating adipogenic gene expression (21) and amplifying lipolysis via stimulating the expression of lipolytic genes (21) are the other possible mechanisms reported for the hypolipidemic properties of L-carnitine.

A very recent meta-analysis concluded that synbiotic supplementation could significantly amend TG, TC, LDL-C, and HDL-C (28). The potential mechanisms for the lipid-ameliorating effects of synbiotics have been reported to be predominantly mediated through their gut microbiota-modulating effects (24, 25), leading to augmented SCFA production and improvement of gut barrier function (25). In this regard, various mechanisms are anticipated, including diminishing cholesterol synthesis via hindering the activity of HMG-CoA reductase (71), reducing intestinal cholesterol absorption concomitant with augmented fecal emission (72), enzymatic deconjugation of bile salts and higher excretion of bile acids (73), and competing with cholesterol for intestinal absorption (74). Based on evidence, synbiotics may confer their hypotriglyceridemic effects through impeding triglyceride absorption from the gut, via stimulating GLP-1 levels (75), stimulating the secretion of FIAF, by which inhibiting endothelial LPL, which may lead to decreased release of triglycerides from circulating chylomicrons and very-low-density lipoprotein (VLDL) (76), and lowering TG levels through reducing hepatic *de novo* lipogenesis, induced by carbohydrate-responsive element-binding protein (ChREBP) and sterol regulatory element-binding protein (SREBP) (76). Also, hindering pro-inflammatory pathways triggered by Toll-like receptor-LPS (TLR-LPS) and prohibiting Toll-like-receptor-4 (TLR4) activation (28, 29), decreasing inflammatory cytokine production and consequently reducing hepatic triglyceride synthesis through attenuating insulin resistance (29), are reported as potential mechanisms.

The present study showed that L-carnitine + synbiotic co-supplementation resulted in a significant amelioration in all glycemic parameters including FBS, insulin, HOMA-IR, and QUICKI, compared with L-carnitine + placebo monotherapy, even when adjusted for possible confounding factors. Additionally, either L-carnitine + synbiotic or L-carnitine + placebo supplementation led to a remarkable intragroup amendment in glycemic parameters, except for FBS in the L-carnitine + placebo group. Considering that both carnitine and synbiotics are perceived to induce beneficial effects on glycemic indices (10, 77), hence, significant intergroup differences in glycemic parameters might have been mediated through the plausible complementary or cumulative effects of L-carnitine + synbiotic co-administration, which led to a greater amelioration in glycemic indices in the co-supplemented arm, or conceivably, could be attributed to the greater weight reduction in the co-supplemented group (78).

Various clinical trials have demonstrated the promising effects of L-carnitine on insulin sensitivity and/or glucose tolerance (10). Our findings were in accordance with the results of a recent meta-analysis, implying the ameliorating impacts of L-carnitine on insulin, FBG, and HOMA-IR (13). Additionally, another meta-analysis of 24 RCTs in patients having cardiovascular risk factors implied the beneficial impacts of L-carnitine on FBG and HOMA-IR (16).

Several mechanisms are suggested for the beneficial impacts of L-carnitine on glucose homeostasis, including improving insulin sensitivity, enhancing glucose utilization, modulating the gluconeogenic and glycolytic enzymes expression, altering the insulin signaling cascade gene expression, and stimulating the insulin-like growth factor 1 (IGF-1) axis and IGF-1 signaling cascade (10). L-carnitine is reported to enhance insulin sensitivity, through augmenting long-chain acyl-CoA ß-oxidation (79), since within cellular aggregation of acyl-CoA derivates is stated to be involved in impairing insulin signaling, inducing insulin resistance in the heart and skeletal muscle (10, 17). Additionally, L-carnitine is supposed to participate in increasing glucose consumption via upregulating PDHC activity (10, 79), also modulating the intramitochondrial acetyl-CoA/CoA ratio (10). Furthermore, L-carnitine may also positively contribute to glucose homeostasis, through downregulating the gluconeogenic enzymes expression, including phosphoenolpyruvate carboxykinase (PCK1) and fructose-1,6-bisphosphatase isozyme 2 (FBP2), while upregulating the glycolytic enzymes expression, e.g., pyruvate kinase and glucokinase (10). Finally, modification of the expression of genes involved in the insulin signaling cascade and activation of the IGF-1 signaling pathway have been suggested as putative mechanisms for improving glucose tolerance (10).

It has been clarified in several clinical trials that pro/pre/synbiotics might improve glucose homeostasis features (77, 80). Synbiotic supplementation in metabolic syndrome patients significantly improved insulin, FBS, QUICKI, and HOMA-IR (80). Moreover, a meta-analysis on diabetic patients, indicated that synbiotic supplementation markedly ameliorated FPG, insulin, HOMA-IR, and QUICKI, supporting our findings as well (81). However, another meta-analysis revealed that synbiotic supplementation in individuals with overweight or obesity merely decreased fasting insulin without any significant effects on other glycemic indices (29). Perhaps, the discrepancy in the results could be due to the diversity in formulations, dosages, or duration of supplementation, as well as different health conditions or the ethnic groups of the subjects (82).

The putative mechanisms for the ameliorating effects of synbiotics on glycemic status principally mediated through amplifying SCFA production could be considered as four principal issues, namely, ameliorating glucose homeostasis, attenuating insulin resistance, amending gut integrity, and hindering proinflammatory signaling pathways (31, 33, 34). An emerging body of evidence supports the impact of SCFAs on improving glucose homeostasis, predominantly signaled through GPCRs, mainly GPR43 (FFAR2) and GPR41 (FFAR3) (31, 33). It has been reported that SCFAs might regulate hepatic glucose homeostasis, through activating AMPK, involving PPAR-γ-mediated effects on gluconeogenesis (31). Furthermore, SCFAs might increase anorectic gut hormone secretion, PYY and GLP-1, which may enhance glucose disposal and decrease insulin resistance (25). Based on evidence, SCFAs might suspend gastric emptying, probably via PYY, leading to hindered glucose release into the bloodstream and reduced postprandial glucose concentrations (61). Moreover, synbiotics have been reported to improve gut integrity via SCFA production, by which inhibiting the TLR4–LPS complex-triggered proinflammatory cascade, hampering proinflammatory cytokine signaling pathways, such as nuclear factor kappa B (NF-κB), thereby decreasing insulin resistance (31, 34).

Collectively, mounting data support the ameliorating impacts of either synbiotics or L-carnitine on obesity, lipid profile, or glycemic markers although distinctions in metabolic modifying agents, such as the participants’ ethnic group, genotype, health condition, dietary intake, physical activity, and baseline values of biological markers, along with formulation, dose, and supplementation length, might have resulted in diverse reports. Furthermore, given the aforementioned purported mechanisms for the improving effects of L-carnitine/synbiotics on weight or metabolic parameters, it could be conceived that concomitant supplementation of L-carnitine and synbiotics may confer more pronounced impacts on these indicators, potentially through simultaneous modulation of energy homeostasis (8, 30), increasing fatty acid oxidation (8, 30), reducing adipogenesis or stimulating adipocyte lipolysis (21, 30), alleviating insulin resistance and glucose homeostasis (10, 31, 33), or attenuating systemic inflammation (56, 57), in a complementary or synergistic manner probably mediated through concurrent targeting of metabolic/inflammatory signaling pathways including CPT-Ia (20, 21), PDHC (8, 19), PPAR-γ (21, 31, 37), AMPK (30, 37), or NF-κB (31, 34).

### Strengths and limitations

As far as we know, this clinical trial appears to be the first to compare the impacts of co-supplementation of L-carnitine + synbiotic vs. L-carnitine single prescription on the anthropometric and metabolic responses among women with obesity. The major strength of the present study was the participants’ recruitment criteria, as it was performed on non-menopause, healthy women with obesity, not taking any medications, thus eliminating the possible confounding factor effects. Moreover, supplementation with a multistrain/multispecies synbiotic might be the other privilege of this research; based on evidence, multistrain pro- or synbiotics appear to be more efficient compared with single strains, due to the plausible synergistic or complementary interactivity among diverse species/strains (25, 81). Also, a small dropout rate and a high level of participants’ adherence (over 90%) were the other positive points.

Nevertheless, this study had some limitations, including a relatively short intervention duration. Furthermore, not including a third intervention group receiving synbiotic plus placebo, could be considered as the other limitation of this study. Further longer duration studies evaluating the gut microbiota composition in a three-group setting are recommended.

## Conclusion

Collectively, the current study implied that supplementation of multistrain/multispecies synbiotic (250 mg/day) concomitant with L-carnitine (2 × 500 mg/day) for 8 weeks resulted in greater amendment in anthropometric and glycemic indices, and HDL-C in healthy female individuals with obesity without any severe side effects, indicating that L-carnitine + synbiotic co-supplementation seems to be more beneficial to ameliorate the mentioned parameters, compared with L-carnitine individual therapy.

In conclusion, our findings suggest that co-administration of L-carnitine and synbiotic may be an encouraging therapeutic strategy for obesity and related cardiometabolic complications, possibly due to simultaneous targeting of multiple metabolic pathways, augmenting their bioavailability/function through potential complementary or synergistic mechanisms. Moreover, co-encapsulation of synbiotics and L-carnitine as a single microcapsule could be considered as a conceivable delivery system to enhance the stability and efficacy of synbiotics as well as to reduce the cost of the final product.

Further mechanistic investigations are warranted to clarify the exact mechanisms mediating the synergistic effect of L-carnitine and synbiotic combined therapy on weight and metabolic parameters.

## Data availability statement

The original contributions presented in the study are included in the article/**Supplementary Material**. Further inquiries can be directed to the corresponding author.

## Ethics statement

The study was performed in accordance with the ethical standards of the Declaration of Helsinki and its later amendments. The study protocol was approved by the Medical Ethics Committee of Tabriz University of Medical Sciences (IR.TBZMED.REC.1396.747). All participants provided their written informed consent to participate in this study.

## Author contributions

FF and RM designed the research and contributed to the conception of the project, development of the overall research plan, and study oversight. FF collected the data, drafted the manuscript, and analyzed and interpreted the data. RM supervised the data interpretation, appraised the article critically, and gave final approval. All authors contributed to the article and approved the submitted version.

## Glossary

ACO: acyl coenzyme A oxidase
AMPK: adenosine monophosphate-activated protein kinase
ANCOVA: analysis of covariance
aP2: adipose-specific fatty acid-binding protein
ARR: absolute risk reduction
BAT: brown adipose tissue
BMI: body mass index
BSH: bile salt hydrolase
CFU: colony-forming unit
Chol: cholesterol
ChREBP: carbohydrate-responsive element-binding protein
CI: confidence interval
CPT-Ia: carnitine palmitoyltransferase Ia
ELISA: enzyme-linked immunosorbent assay
FBG: fasting blood glucose
FBS: fasting blood sugar
FBP2: fructose-1,6-bisphosphatase isozyme 2
FFAs: free fatty acids
FFAR3: free fatty acid receptor 3
FFAR2: free fatty acid receptor 2
FIAF: fasting-induced adipose factor
FOS: fructo-oligosaccharide
FXR: farnesoid X receptor
GLP-1: glucagon-like peptide-1
GLP-2: glucagon-like peptide-2
GPR41: G protein-coupled receptor 41
GPR43: G protein-coupled receptor 43
GPCRs: G protein-coupled receptors
HC: hip circumference
HDL-C: high-density lipoprotein-cholesterol
HMG-CoA: hydroxymethylglutaryl CoA reductase
HOMA-IR: homeostatic model assessment of insulin resistance
HSL: hormone-sensitive lipase
IGF-1: insulin-like growth factor 1
IPAQ-SF: International Physical Activity Questionnaire-Short Form
ITT: intention-to-treat
LDL-C: low-density lipoprotein-cholesterol
LPL: lipoprotein lipase
LPS: lipopolysaccharide
METs: metabolic equivalent of tasks
NF-κB: nuclear factor kappa B
NHLBI: National Heart, Lung, and Blood Institute
NNT: number needed to treat
NSAIDs: non-steroidal anti-inflammatory drugs
OTC: over the counter
PC: percent of changes
PCK1: phosphoenolpyruvate carboxykinase
PDHC: pyruvate dehydrogenase complex
PPAR-γ: peroxisome proliferator-activated receptor-gamma
PGC-1α: peroxisome proliferator-activated receptor-gamma coactivator 1α
PYY: peptide YY
QUICKI: quantitative insulin sensitivity check index
RAS: random allocation software
RCT: randomized clinical trial
REE: resting energy expenditure
SCFAs: short-chain fatty acids
SD: standard deviation
SREBP: sterol regulatory element-binding protein
TC: total cholesterol
TG: triglyceride
TGR5: Takeda G protein-coupled bile acid receptor 5
TLR-LPS: Toll-like receptor-LPS
TLR4: Toll-like receptor 4
UCP-1: uncoupling protein-1
VLDL: very low-density lipoprotein
WAT: white adipose tissue
WC: waist circumference
WHR: waist to hip ratio
