# Supplementary material for: Ameliorating effects of L-carnitine and synbiotic co-supplementation on anthropometric measures and cardiometabolic traits in women with obesity: a randomized controlled clinical trial
Source: Front Endocrinol (Lausanne). 2023 Oct 18;14:1237882. doi: 10.3389/fendo.2023.1237882 (PMC10622781; doi:10.3389/fendo.2023.1237882)
Supplement: Supplementary file 1 [file DataSheet_1.pdf]

Supplementary Material

Ameliorating effects of L-carnitine and synbiotic co-supplementation on anthropometric measures and cardiometabolic traits in women with obesity: a randomized controlled clinical trial

Farnoush Fallah[1], **Reza Mahdavi**[2]*

[1]Student Research Committee, Nutrition Research Center, Tabriz University of Medical Sciences, Tabriz, Iran. E-mail: fallahfarnoush@gmail.com

[*2]Nutrition Research Center, Department of Biochemistry and Diet Therapy, Faculty of Nutrition and Food Sciences, Tabriz University of Medical Sciences, Tabriz, Iran. E-mail: mahdavir@tbzmed.ac.ir

*** Correspondence:**

Reza Mahdavi

Nutrition Research Center, Department of Biochemistry and Diet Therapy, Faculty of Nutrition and Food Sciences, Tabriz University of Medical Sciences, Tabriz, Iran. E-mail: mahdavir@tbzmed.ac.ir

1. Supplementary Tables

1.1. CONSORT checklist for reporting a randomized clinical trial

1.2. Study results based on Intention to treat (ITT) statistical analysis (n=46)

CONSORT checklist for reporting a randomised clinical trial

Section/Topic	Item No	Checklist item	Reported on page No
**Title and abstract**			
	1a	Identification as a randomized trial in the title	Title page
	1b	Structured summary of trial design, methods, results, and conclusions	Abstract, Page 2
**Introduction**			
Background and objectives	2a	Scientific background and explanation of rationale	Pages 3 & 4
	2b	Specific objectives or hypotheses	Page 4
**Methods**			
Trial design	3a	Description of trial design (such as parallel, factorial) including allocation ratio	Pages 4 & 5
	3b	Important changes to methods after trial commencement (such as eligibility criteria), with reasons	No Changes
Participants	4a	Eligibility criteria for participants	Page 4
	4b	Settings and locations where the data were collected	Page 4
Interventions	5	The interventions for each group with sufficient details to allow replication, including how and when they were actually administered	Page 5
Outcomes	6a	Completely defined pre-specified primary and secondary outcome measures, including how and when they were assessed	Pages 5, 6, 7
	6b	Any changes to trial outcomes after the trial commenced, with reasons	No Changes
Sample size	7a	How sample size was determined	Page 5
	7b	When applicable, explanation of any interim analyses and stopping guidelines	Not Applicable
Randomization:			
Sequence generation	8a	Method used to generate the random allocation sequence	Page 5
	8b	Type of randomization; details of any restriction (such as blocking and block size)	Page 5
Allocation Concealment mechanism	9	Mechanism used to implement the random allocation sequence (such as sequentially numbered containers), describing any steps taken to conceal the sequence until interventions were assigned	Page 5
Implementation	10	Who generated the random allocation sequence, who enrolled participants, and who assigned participants to interventions	Page 5

Blinding	11a	If done, who was blinded after assignment to interventions (for example, participants, care providers, those assessing outcomes) and how	Page 5
	11b	If relevant, description of the similarity of interventions	Page 5
Statistical methods	12a	Statistical methods used to compare groups for primary and secondary outcomes	Page 6
	12b	Methods for additional analyses, such as subgroup analyses and adjusted analyses	Page 6
Participant flow (a diagram is strongly recommended)	13a	For each group, the numbers of participants who were randomly assigned, received intended treatment, and were analyzed for the primary outcome	Page 6, Fig 1
	13b	For each group, losses, and exclusions after randomization, together with reasons	Page 6, Fig 1
Recruitment	14a	Dates defining the periods of recruitment and follow-up	Page 4
	14b	Why the trial ended or was stopped	Not Applicable
Baseline data	15	A table showing baseline demographic and clinical characteristics for each group	Table1, page 20
Numbers analyzed	16	For each group, number of participants (denominator) included in each analysis and whether the analysis was by original assigned groups	Fig 1
Outcomes and estimation	17a	For each primary and secondary outcome, results for each group, and the estimated effect size and its precision (such as 95% confidence interval)	Tables 2,3,4 Pages 21-23
	17b	For binary outcomes, presentation of both absolute and relative effect sizes is recommended	Not Applicable
Ancillary analyses	18	Results of any other analyses performed, including subgroup analyses and adjusted analyses, distinguishing pre-specified from exploratory	Tables 2,3,4 Pages 21-23
Harms	19	All important harms or unintended effects in each group (for specific guidance see CONSORT for harms)	No harms
**Discussion**			
Limitations	20	Trial limitations, addressing sources of potential bias, imprecision, and, if relevant, multiplicity of analyses	Page 12
Generalizability	21	Generalizability (external validity, applicability) of the trial findings	Page 12
Interpretation	22	Interpretation consistent with results, balancing benefits, and harms, and considering other relevant evidence	Pages 7-12
**Other information**			
Registration	23	Registration number and name of trial registry	Abstract, Page 4
Protocol	24	Where the full trial protocol can be accessed, if available	Abstract, Page 4
Funding	25	Sources of funding and other support (such as supply of drugs), role of funders	Page 14

Table 1 Baseline characteristics of the study participants

	L-carnitine+Synbiotic (n=23)	L-carnitine+Placebo (n=23)	*P*
**Age** (years)	38.39 (6.30)	38.00 (7.29)	0.847 [a]
**Weight** (kg)	84.04 (8.67)	85.01 (7.70)	0.691 [a]
**Height** (cm)	160.56 (6.02)	159.95 (6.64)	0.746 [a]
**BMI** (kg m^{-2})	32.59 (2.02)	33.22 (1.71)	0.262 [a]
**PA (METs)** (METs-minutes per week)	776.95 (276.83)	715.56 (300.07)	0.475 [a]
**Marital status**			
Single	3 (13.00)	4 (17.4)	0.715 [b]
Married	19 (82.6)	19 (82.6)	
Divorced or Widow	1 (4.3)	0 (0.0)	
**Education**			
Illiterate	0 (0.0)	1 (4.3)	0.589 [b]
Diploma and lower	11 (47.8)	12 (52.2)	
Bachelors or higher	12 (52.2)	10 (43.5)	
**Occupation**			
Housewife	11 (47.8)	16 (69.6)	0.172 [b]
Employee	11 (47.8)	7 (30.4)	
Self-employed	1 (4.3)	0 (0.0)	

Numerical data are expressed as mean (SD) and categorical variables as number (%).
a p-value based on Independent-samples' t-test
b p-value based on Chi-square test

Table 2 Dietary intakes and physical activity of participants throughout the study

	L-carnitine+Synbiotic (n=23)	L-carnitine+Placebo (n=23)	MD (95% CI) (between-groups)	*P*
**Energy** (Kcal d^{-1})				
Baseline	2390.21 (660.67)	2086.30 (450.88)	303.91 (-32.21, 640.04)	0.075 [b]
After 4 weeks	2322.78 (590.99)	1974.21 (446.28)	76.58 (-20.39, 173.56) [f]	0.119 [c]
End	2300.91 (541.14)	1950.73 (376.92)	147.19 (-32.45, 326.84) [f]	0.106 [c]
P [a]	0.161	0.166		
**Protein** (g d^{-1})				
Baseline	72.41 (17.43)	69.35 (19.54)	3.06 (-7.94, 14.07)	0.578 [b]
After 4 weeks	69.66 (13.59)	62.76 (13.54)	4.71 (-1.68, 11.12) [f]	0.144 [d]
End	73.47 (14.91)	64.23 (21.82)	8.13 (-0.76, 17.02) [f]	0.072 [d]
P [a]	0.377	0.199		
**Carbohydrates** (g d^{-1})				
Baseline	313.25 (104.96)	263.47 (79.08)	49.78 (-5.44, 105.00)	0.076 [b]
After 4 weeks	302.84 (94.28)	243.93 (63.37)	10.63 (-9.46, 30.73) [f]	0.292 [d]
End	298.59 (117.49)	250.08 (66.57)	1.45 (-27.60, 30.52) [f]	0.920 [d]
P [a]	0.249	0.125		
**Fat** (g d^{-1})				
Baseline	90.04 (24.25)	88.16 (27.07)	1.88 (-13.39, 17.15)	0.805 [b]
After 4 weeks	87.48 (18.11)	81.15 (17.94)	4.80 (-0.92, 10.53) [f]	0.098 [d]
End	88.21 (23.24)	77.89 (23.74)	7.26 (-2.22, 16.76) [f]	0.130 [d]
P [a]	0.694	0.189		
**Dietary fiber** (g d^{-1})				
Baseline	11.66 (4.74)	10.12 (3.99)	1.53 (-1.06, 4.14)	0.241[b]
After 4 weeks	11.85 (4.60)	11.66 (2.57)	-0.78 (-2.44, 0.87) [f]	0.346 [d]
End	10.83 (4.74)	10.90 (5.36)	-1.16 (-3.78, 1.45) [f]	0.374 [d]
P [a]	0.268	0.237		
**PA (METs)** (METs-minutes per week)				
Baseline	776.95 (276.83)	715.56 (300.07)	61.39 (-110.17, 232.95)	0.475 [b]
After 4 weeks	803.47 (256.76)	716.08 (324.85)	26.28 (-7.76, 60.34) [f]	0.127 [e]
End	784.56 (282.65)	719.65 (312.80)	3.09 (-36.86, 43.05) [f]	0.877 [e]
P [a]	0.230	0.870		

METs, metabolic equivalents (MET-minutes/week); MD, mean difference; CI, confidence interval; Data are presented as mean (SD) and mean difference (95% CI).
[a] p-value based on Repeated measures analysis of variance (RM-ANOVA) (comparison of data with more than 2 measurements within the groups post-intervention); [b] p-value based on Independent samples' t-test (comparison of data between the groups at the baseline); [c] p-value based on Analysis of covariance (ANCOVA) (comparison of data between the groups post-intervention, adjusted for baseline values and changes in physical activity); [d] p-value based on Analysis of covariance (ANCOVA) (comparison of data between the groups post-intervention, adjusted for baseline values, changes in physical activity and energy intakes); [e] p-value based on ANCOVA (comparison of data between the groups post-intervention, adjusted for baseline values); [f] Absolute effect size (95% CI) based on the mentioned ANCOVA models; Bold values indicate statistically significant differences ($p<0.05$).

Table 3 Changes in anthropometric indices of participants at baseline and end of the study

	**L-carnitine+Synbiotic (n=23)**	**L-carnitine+Placebo (n=23)**	**MD (95% CI) (between-groups)**	***P***
**Weight** (kg)				
Baseline	84.04 (8.67)	85.01 (7.70)	-0.96 (-5.84, 3.90)	0.691 [b]
End	79.81 (7.42)	84.08 (7.28)	-3.41 (-4.33, -2.49) [d]	**< 0.001** [c]
**MD (95% CI)** (within-groups)	-4.23 (-5.18, -3.26)	-0.92 (-1.53, -0.31)		
P [a]	**< 0.001**	**0.005**		
**BMI** (kg m^{-2})				
Baseline	32.59 (2.02)	33.22 (1.71)	-0.62 (-1.74, 0.48)	0.262 [b]
End	30.98 (1.88)	32.86 (1.55)	-1.33 (-1.70, -0.96) [d]	**< 0.001** [c]
**MD (95% CI)** (within-groups)	-1.61 (-1.94, -1.28)	-0.35 (-0.59, -0.12)		
P [a]	**< 0.001**	**0.004**		
**WC** (cm)				
Baseline	101.39 (8.82)	103.95 (7.83)	-2.56 (-7.52, 2.39)	0.303 [b]
End	92.97 (7.37)	97.86 (6.32)	-3.08 (-5.36, -0.80) [d]	**0.009** [c]
**MD (95% CI)** (within-groups)	-8.41 (-10.87, -5.94)	-6.08 (-7.38, -4.79)		
P [a]	**< 0.001**	**< 0.001**		
**HC** (cm)				
Baseline	116.30 (5.77)	117.54 (5.89)	-1.23 (-4.70, 2.22)	0.475 [b]
End	110.04 (4.70)	112.67 (5.40)	-1.71 (-3.20, -0.21) [d]	**0.026** [c]
**MD (95% CI)** (within-groups)	-6.26 (-7.14, -5.37)	-4.86 (-6.38, -3.35)		
P [a]	**< 0.001**	**< 0.001**		
**WHR**				
Baseline	0.87 (0.06)	0.88 (0.06)	-0.01 (-0.05, 0.02)	0.479 [b]
End	0.84 (0.06)	0.87 (0.06)	-0.01 (-0.03, 0.006) [d]	0.146 [c]
**MD (95% CI)** (within-groups)	-0.02 (-0.04, -0.009)	-0.01 (-0.02, -0.003)		
P [a]	**0.006**	**0.010**		
**NC**				
Baseline	38.33 (1.62)	38.81 (1.25)	-0.47 (-1.34, 0.38)	0.271 [b]
End	36.75 (1.57)	38.43 (1.05)	-1.28 (-1.63, -0.93) [d]	**< 0.001** [c]
**MD (95% CI)** (within-groups)	-1.58 (-1.88, -1.28)	-0.37 (-0.61, -0.14)		
P [a]	**< 0.001**	**0.003**		

BMI, body mass index; WC, waist circumference; HC, hip circumference; WHR, waist to hip ratio; **NC**, **neck** circumference**;** MD, mean difference; CI, confidence interval**;** Data are presented as mean (SD) and mean difference (95% CI).

[a] *p*-value based on Paired samples' *t*-test (comparison of data within the groups post-intervention); [b] *p*-value based on Independent samples' *t*-test (comparison of data between the groups at the baseline); [c] *p*-value based on Analysis of covariance (ANCOVA) (comparison of data between the groups post-intervention, adjusted for baseline values, changes in physical activity and energy intakes); [d] Absolute effect size (95% CI) based on the mentioned ANCOVA models; Bold values indicate statistically significant differences (p<0.05).

Table 4 Changes in lipid profile and glycemic indices of participants at baseline and end of the study

	**L-carnitine+Synbiotic (n=23)**	**L-carnitine+Placebo (n=23)**	**MD (95% CI) (between-groups)**	***P***
**TG** (mg dl^{-1})				
Baseline	124.73 (39.21)	111.56 (35.05)	13.17 (-8.92, 35.27)	0.236 [b]
End	101.30 (26.26)	101.82 (28.13)	-6.99 (-22.07, 8.07) [d]	0.354 [c]
**MD (95% CI)** (**within-group**	-23.43 (-35.01, -11.85)	-9.73 (-20.11, 0.64)		
P [a]	**< 0.001**	0.065		
**TC** (mg dl^{-1})				
Baseline	189.34 (25.38)	191.47 (38.28)	-2.13 (-21.51, 17.25)	0.825 [b]
End	145.47 (18.08)	153.86 (27.53)	-5.94 (-20.56, 8.67) [d]	0.416 [c]
**MD (95% CI)** (**within-group**	-43.86 (-53.62, -34.11)	-37.60 (-49.16, -26.04)		
P [a]	**< 0.001**	**< 0.001**		
**LDL-C** (mg dl^{-1})				
Baseline	112.17 (22.98)	125.56 (30.78)	13.39 (-29.53, 2.75)	0.102 [b]
End	84.08 (15.27)	92.65 (24.09)	-1.00 (-14.14, 12.14) [d]	0.878 [c]
**MD (95% CI)** (**within-group**	-28.08 (-36.61, -19.55)	-32.91 (-42.56, -23.26)		
P [a]	**< 0.001**	**< 0.001**		
**HDL-C** (mg dl^{-1})				
Baseline	41.08 (8.65)	40.86 (6.98)	0.21 (-4.45, 4.89)	0.926 [b]
End	51.69 (11.10)	43.73 (7.25)	7.01 (2.42, 11.60) [d]	**0.004** [c]
**MD (95% CI)** (**within-group**	10.60 (8.19, 13.01)	2.86 (0.43, 5.30)		
P [a]	**< 0.001**	**0.023**		
**FBS** (mg dl^{-1})				
Baseline	89.95 (8.88)	85.82 (11.28)	4.13 (-1.90, 10.16)	0.175 [b]
End	75.65 (4.75)	81.73 (8.47)	-7.65 (-13.10, -2.19) [d]	**0.007** [c]
**MD (95% CI)** (**within-group**	-14.30 (-17.06, -11.54)	-4.08 (-9.53, 1.35)		
P [a]	**< 0.001**	0.134		
**Insulin** (µIU ml^{-1})				
Baseline	19.83 (12.69)	20.82 (10.95)	- 0.98 (-8.03, 6.05)	0.779 [b]
End	10.50 (3.19)	14.34 (5.16)	-3.52 (-7.06, 0.02) [d]	0.051 [c]
**MD (95% CI)** (**within-group**	-9.32 (-14.46, -4.18)	-6.48 (-1.71, -1.25)		
P [a]	**0.001**	**0.017**		
**HOMA-IR**				
Baseline	4.37 (2.79)	4.47 (2.60)	-0.09 (-1.70, 1.50)	0.903 [b]
End	1.97 (0.66)	2.93 (1.15)	-0.88 (-1.63, -0.12) [d]	**0.023** [c]
**MD (95% CI)** (**within-group**	-2.40 (-3.52, -1.27)	-1.53 (-2.69, -0.37)		
P [a]	**< 0.001**	**0.012**		
**QUICKI**				
Baseline	0.31 (0.03)	0.31 (0.02)	0.003 (-0.01, 0.02)	0.696 [b]
End	0.34 (0.01)	0.32 (0.01)	0.01 (0.003, 0.031) [d]	**0.017** [c]
**MD (95% CI)** (**within-group**	0.02 (0.01, 0.04)	0.01 (0.002, 0.023)		
P [a]	**< 0.001**	**0.018**		

TG, triglyceride; TC, total cholesterol; LDL-C, low-density lipoprotein cholesterol; HDL-C, high-density lipoprotein cholesterol; FBS, fasting blood sugar; HOMA-IR, homeostasis model assessment of insulin resistance; QUICKI, quantitative insulin sensitivity check index; MD, mean difference; CI, confidence interval; Data are presented as mean (SD) and mean difference (95% CI).

[a] *p*-value based on Paired samples' *t*-test (comparison of data within the groups post-intervention); [b] *p*-value based on Independent samples' *t*-test (comparison of data between the groups at the baseline); [c] *p*-value based on Analysis of covariance (ANCOVA) (comparison of data between the groups post-intervention, adjusted for baseline values, changes in physical activity, energy intakes, and BMI); [d] Absolute effect size (95% CI) based on the mentioned ANCOVA models; Bold values indicate statistically significant differences ($p<0.05$).
